# Relationship between Depression with Physical Activity and Obesity in Older Diabetes Patients: Inflammation as a Mediator

**DOI:** 10.3390/nu14194200

**Published:** 2022-10-09

**Authors:** Jui-Hua Huang, Ren-Hau Li, Leih-Ching Tsai

**Affiliations:** 1 Department of Golden-Ager Industry Management, Chaoyang University of Technology, Taichung 413, Taiwan; 2 Department of Psychology, Chung Shan Medical University, Taichung 402, Taiwan; 3 Division of Endocrine and Metabolism, Department of Internal Medicine, Erlin-Branch, Changhua Christian Hospital, Changhua 52600, Taiwan

**Keywords:** depression, inflammation, obesity, physical activity, older diabetes patient

## Abstract

Obesity and physical activity (PA) may affect inflammation and are also related to depression. This study aimed to explore the association between depression, obesity, and PA in older diabetes patients mediated by inflammation. We conducted a cross-sectional study with 197 elderly diabetes patients (≥65 y/o). Participants were interviewed to gather demographic and lifestyle data. Assessment of depression was based on the Diagnostic and Statistical Manual of Mental Disorders, Fourth Edition (DSM-IV) criteria. High-sensitivity C-reactive protein was used as a marker of inflammation. Participants with a body mass index (kg/m^2^) ≥ 27 were considered to be obese. Our data indicated that among all participants with (*n* = 57) and without (*n* = 140) depression, older diabetes patients with depression had a lower intake of energy and protein and a lower prevalence of smoking and alcohol consumption than those without depression (*p* < 0.05). We also found that inflammation may be a partial mediator in the relationship between obesity and depression, and a significant mediator between PA and depression. Additionally, a regression model of obesity and PA showed that PA was a significant predictor of inflammation. However, the association between obesity and inflammation was not significant. When obesity, PA, and inflammation were included in a regression model together, inflammation significantly predicted depression (OR = 4.18, *p* = 0.004). The association between obesity and depression was also significant (OR = 2.45, *p* = 0.038). However, the association between PA and depression was not significant, and the mediating effect of inflammation was significant according to the Sobel test (z = −2.01, *p* = 0.045). In conclusion, the beneficial effects of PA may lower levels of inflammation produced by obesity, thus reducing inflammatory effects that may be related to depression. Overall, inflammation may mediate the relationship between depression and PA in older diabetes patients.

## 1. Introduction

Diabetes is a highly prevalent chronic disease [1], and elderly patients with diabetes have a higher rate of depression [2,3]. Inflammation may play a critical role in developing depression [4]. Inflammatory molecules may access the brain and influence neurotransmitter systems related to depression [4]. Adipose tissue is a plentiful source of inflammatory molecules [5]. The relationship between obesity and depression has also previously been described [5]. In addition, lifestyle factors such as physical activity (PA) may impact inflammation and are also associated with the development of depression [6,7,8,9]. However, the inter-relationships between depression, inflammation, obesity, and PA are unclear.

Several studies have indicated that inflammation and depression may be related [10,11,12]. A meta-analysis suggested that patients with depression showed elevated levels of pro-inflammatory cytokines and C-reactive protein (CRP) [10]. As for the inflammatory source, adipose tissue is the primary source of inflammatory molecules [13]. Obesity predisposes the body to a pro-inflammatory state by adipose tissue releasing inflammatory mediators, such as interleukin 6, which triggers hepatocytes to synthesize and secrete CRP [13]. Moreover, a meta-analysis found that obesity may correlate with depression [14]. Cross-sectional study data revealed that elevated levels of CRP were relevant to a high prevalence of depression in diabetes patients with high BMIs [12]. Nevertheless, the degree of association between obesity and depression in older diabetes patients, mediated through inflammation, remains debatable.

In addition, growing evidence is indicating that PA levels are associated with inflammation and depression [9,15,16,17]. A recent study demonstrated that PA was inversely associated with inflammatory markers in people with type 2 diabetes [15]. Raised levels of CRP were associated with inactivity [16]. Moreover, some studies have shown the association between PA and depression. Song et al. found that adults with depression were less active than adults without depression [9]. Vallance et al. identified a lower risk of depression related to increased PA levels and decreased sedentary behavior [17]. However, quantifying these positive effects of PA on depression, mediated by inflammation, in older diabetes patients remains to be determined.

Inflammation may be as a mediating pathway in the development of depression. Obesity and PA may affect levels of inflammation and are also related to the development of depression [9,13,14,16]. Very few studies have examined the relationship between obesity, PA, depression, and the potential mediating role of inflammation in older diabetes patients. Further clarification of these associations is required. According to Baron and Kenny’s four-step approach [18,19], we proposed the following a priori hypotheses: (1) PA or obesity will significantly predict depression; (2) PA or obesity will significantly predict inflammation; (3) inflammation will significantly predict depression; and (4) after adjusting for inflammation, the association between PA or obesity and depression will be reduced (partial mediation by inflammation) or will no longer be significant (complete mediation by inflammation). In addition, the Sobel test was used as a supplementary technique [20].

## 2. Materials and Methods

### 2.1. Ethics Statement

The IRB approved this study of the Changhua Christian Hospital in Taiwan (CCHIRB#090419).

### 2.2. Study Sample

The sample size of this study was estimated according to sample size calculations for cross-sectional studies/surveys [21]. Previously published studies have suggested that actual prevalence of depression may be between 13 and 17% for the elderly populations of Taiwan [22,23,24]. We wanted to calculate this sample size with the precision/absolute error of 5% and type 1 error of 5%. So, sample size of the present study ranged approximately between 174 and 217.

After obtaining written informed consent, 236 elderly diabetes patients from a rural area of central Taiwan were enrolled in the present study. The inclusion criteria included those aged 65 years and above, with type 2 diabetes patients for more than six months, no medication change for the past three months and maintaining a stable lifestyle for three months. Exclusion criteria included heart failure, cirrhosis, current malignancy, chronic renal failure, clinically relevant infections (CRP levels > 10 mg/L), or signs of severe deterioration in comprehension or memory. Participant were interviewed to obtain information on personal data, PA, and lifestyle factors through a questionnaire. Assessment of depression was done according to the DSM-IV criteria. Clinical examination included measures of anthropometric and blood biochemical variables. After excluding 39 older diabetes patients who could not provide the necessary information on personal data and lifestyle, 197 older diabetes patients were included in the final analysis.

### 2.3. Assessment of Covariables for Lifestyle Factors

Data on dietary intake were obtained using 24 h recall [25]. Additionally, participants were asked to describe a week of their typical nutritional pattern in the survey to assess daily dietary intake using nutritional analysis software (Professional Edition, version 2001/2003, EKitchen Inc., Taichung, Taiwan), which estimated energy and nutrient intake based on the Taiwan Nutrition Database. Information regarding habits of smoking and alcohol consumption was collected from each question. Smoking habits were classified into three categories: never, former, and current. In addition, participants were classified into the following three conditions regarding alcohol consumption: never consumed, formerly consumed, and currently consuming.

### 2.4. Assessment of Depression

A depression assessment based on the Diagnostic and Statistical Manual of Mental Disorders, Fourth Edition (DSM-IV) criteria, including number and severity of symptoms, duration, and course of illness [26], was administered for all participants. DSM-IV has nine symptoms as follows: (1) depressed mood; (2) markedly diminished interest or pleasure in most or all activities; (3) significant weight loss (or poor appetite) or weight gain; (4) insomnia or hypersomnia; (5) psychomotor retardation; (6) fatigue or loss of energy; (7) feelings of worthlessness or excessive or inappropriate guilt; (8) diminished ability to think or concentrate, or indecisiveness; and (9) recurrent thoughts of death (not just fear of dying) or suicidal ideation, plans, or attempts. The diagnosis of depression was based on the presence of five or more symptoms for ≥2 weeks, including at least one key sign (depressed mood or loss of interest/pleasure). Symptoms must cause significant distress or impairment and no manic or hypomanic behavior, as defined by the American Psychiatric Association [26].

### 2.5. Assessment of Obesity

The anthropometric measurements included height and weight. Body mass index (BMI) was calculated by dividing weight in kilograms by height in meters squared [(kg)/(m^2^)]. A BMI (kg/m^2^) ≥ 27 was considered obese, according to the definition of the Health Promotion Administration, Ministry of Health and Welfare in Taiwan [27].

### 2.6. Assessment of PA Levels

Implementing the method of a Finnish study that had estimated PA levels [28], participants reported their occupational, commuting, and leisure-time PA. Occupational PA was divided into three categories: (1) light: physical work that requires only seated work; (2) moderate: work that requires standing and walking; and (3) active: work that requires walking and lifting or heavy manual labor. Daily commuting PA was categorized into three categories: (1) use of motorized transportation or no work; (2) walking or biking 1–29 min, and (3) walking or biking for more than 30 min. Leisure-time PA was divided into three categories: (1) low: inactive or doing only minor PA; (2) moderate: some moderate-intensity aerobic PA for 150–300 min per week or vigorous aerobic PA for 75–150 min per week; and (3) high: moderate-intensity aerobic PA for more than 300 min per week or vigorous aerobic PA for more than 150 min per week [29]. The three different types of PA and their levels listed above were used to estimate total PA. Total PA was evaluated and classified into the following three levels: (1) low PA, characterized by low levels of occupational, commuting, and leisure-time PA; (2) moderate PA, characterized by only one of the three types of moderate to high PA; and (3) high PA, characterized by two or three types of moderate to high PA.

### 2.7. Biochemical Determinations of Blood and Definition of Inflammation

High-sensitivity C-reactive protein (hsCRP) in circulating blood was measured to determine inflammation levels [30,31]. This inflammatory marker was measured in a hospital medical laboratory (certified ISO15189). The hsCRP (CV < 3.0%) was measured by particle-enhanced turbidimetric immunoassay (Dimension, Siemens, Newark, USA). The hsCRP levels of <1, 1–3, and >3 mg/L represented low, average, and high inflammation, respectively. When inflammation served as a dependent variable, it was categorized as high inflammation (greater than 3 mg/L of hsCRP) and non-high inflammation (the remainders) to perform a binary logistic regression.

Ion-exchange chromatography (BioRad Variant, Hercules, California, USA) was used to measure glycated hemoglobin (HbA1C). In addition, the Taiwan Society of Nephrology recommends the simplified Modification of Diet in Renal Disease (MDRD) formula to calculate the estimated glomerular filtration rate (eGFR). The eGFR (mL/min/1.73 m^2^) (Simplified MDRD formula) = 186 × serum creatinine^−^^1.154^ × Age^−^^0.203^ in men, and 186 × serum creatinine^−^^1.154^ × Age^−^^0.203^ × 0.742 in women [32].

### 2.8. Statistical Analysis

**Figure 1:**
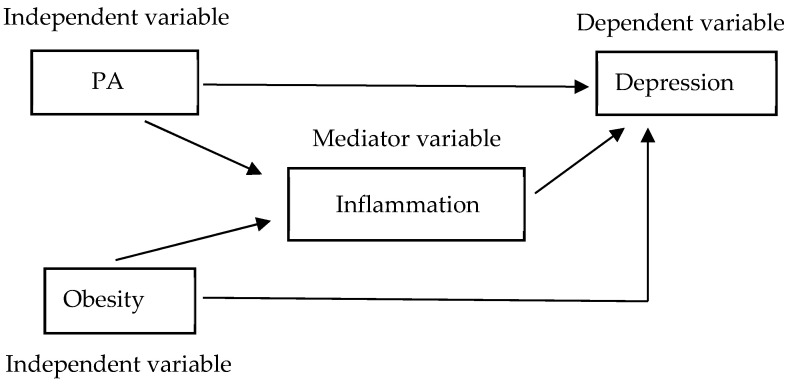
Theoretical model of the present study, based on Baron and Kenny’s four-step approach. Effect of PA and obesity on depression, with inflammation potentially acting as a mediator. There are four criteria required to conclude mediation: (1) the independent variable (PA or obesity) should significantly predict the dependent variable (depression); (2) the independent variable (PA or obesity) should significantly predict the mediating variable (inflammation); (3) the mediating variable (inflammation) should significantly predict the dependent variable (depression); and (4) after adjusting for the mediating variable (inflammation), the association between the independent variable (PA or obesity) and the dependent variable (depression) should be reduced (partial mediation) or should be no longer significant (complete mediation).

A chi-square test was used to analyze categorical variables. When cells had an expected count less than 5, data were analyzed using Fisher’s exact test. Data are presented by number (n) and percent (%). A two-tailed *t*-test (2 groups) was used to analyze the mean comparisons of the continuous dependent variable. Relationships between obesity, PA levels, hsCRP, and depression were assessed using binary logistic regression analysis. In addition, we determined mediation in the relationship between PA, obesity, hsCRP, and depression according to Baron and Kenny’s four-step approach (Figure 1) [18,19]. A mediating effect was calculated by multiplication of its two sub-paths, and a Sobel test was used as a supplementary technique to test it [20]. All statistical procedures were performed using SPSS 22.0 statistical software (SPSS Inc., Chicago, IL, USA), and a *p*-value less than 0.05 was considered statistically significant.

## 3. Results

### 3.1. Characteristics of Older Adults with Type 2 Diabetes According to Depression and Inflammation Levels

A summary of the characteristics of 197 older diabetes patients can be found in Table 1. There were significant differences in gender, energy intake, smoking, and alcohol consumption between the patients with and without depression (*p* < 0.05). The older diabetes patients with depression had lower energy intake and protein intake than those without depression (*p* < 0.05). Furthermore, older diabetes patients with depression had a lower prevalence of smoking and alcohol consumption than those without depression (*p* < 0.05).

There were significant differences in eGFR and smoking among the different inflammation levels (*p* < 0.030). However, inflammation was not significantly associated with personal data, education levels, and other health-related variables.

### 3.2. Prevalence of Depression and Inflammation for Combined PA Levels with Obesity Status

Older diabetes patients were stratified into six subgroups based on PA levels and obesity status: (i) group A had low PA and was obese; (ii) group B had moderate PA and was obese; (iii) group C had high PA and was obese; (iv) group D had low PA and was not obese; (v) group E had moderate PA and was not obese; (vi) group F had high PA and was not obese. The findings showed that combined PA levels with obesity status was correlated with depression and high inflammation (*p* < 0.001 vs. *p* = 0.001), as shown in Table 2. The rate of depression in groups A, B, C, D, E, and F were 63.2, 52.4, 29.4, 40.7, 19.6, and 12.3% (*p* < 0.01), respectively. The rate of high inflammation in groups A, B, C, D, E, and F were 47.4, 28.6, 0, 18.5, 17.9, and 8.8% (*p* = 0.002), respectively.

According to PA levels, there was a significant difference in the prevalence of depression (low: 63.2, moderate: 52.4, high: 29.4%) for older diabetes patients with obesity. According to PA levels, there was also a significant difference in the prevalence of depression (low: 40.7, moderate: 19.6, high: 12.3%) for older diabetes patients who were not obese.

In addition, a similar trend to the above results was observed. According to PA levels, there was a significant difference in the prevalence of high inflammation (low: 47.4, moderate: 28.6, high: 0%) for older diabetes patients with obesity. According to PA levels, there was also a significant difference in the prevalence of inflammation (low: 18.5, moderate: 17.9, high: 8.8%) for older diabetes patients who were not obese.

### 3.3. Analysis of the Relationship between PA, Obesity, Inflammation, and Depression Based on Binary Logistic Regression

Results of the binary logistic regression analyses for the relationships between PA, obesity, inflammation, and depression are summarized in Table 3. We adjusted for confounding factors, including demographic data and health-related lifestyle factors. Obesity, PA, and inflammation were entered into the model, respectively, when adjusting for confounding factors for predicting depression. Obesity, PA, and inflammation were significant predictors of depression, respectively (see model 1). There was an indication of a crude effect whereby a higher PA level was associated with a lower risk of depression (odds ratio, OR = 0.32, *p* = 0.023). Still, obesity (OR = 2.91, *p* = 0.009) and higher hsCRP (OR = 5.14, *p* = 0.001) was associated with a higher risk of depression.

When adjusting for confounding factors in predicting inflammation, obesity and PA were entered into the model, respectively. PA and obesity were both significant associated with inflammation, respectively (see model 2). A higher PA level was associated with a lower risk of inflammation (OR = 0.18, *p* = 0.006), but obesity was associated with a higher risk of inflammation (OR = 2.59, *p* = 0.045).

When adjusting for confounding factors in predicting depression, obesity and inflammation were entered into the model together. Obesity and inflammation still showed statistical significance in predicting depression (see model 3). The odds ratio of associating obesity with the risk of depression changed from 2.91 in model 1 (*p* = 0.009) to 2.54 in model 3 (*p* = 0.031). The mediating effect of inflammation (OR = 4.29, calculated in logits units and transformed to OR) tested significantly on the Sobel test (*z* = 1.97, *p* = 0.049). Therefore, inflammation was a significant partial mediator of the relationship between obesity and depression.

PA and inflammation were entered into the model together when adjusting for confounding factors for predicting depression. Inflammation still showed statistical significance in predicting depression. However, the association between PA and depression was no longer significant (see model 4). The odds ratio of associating PA to the risk of depression changed from 0.32 in model 1 (*p* = 0.023) to 0.43 (*p* = 0.109) in model 4. The mediating effect of inflammation (OR = 0.07, calculated in logits units and transformed to OR) tested significantly on the Sobel test (*z* = −2.00, *p* = 0.045). This indicated that inflammation was a significant mediator in the relationship between PA and depression.

When adjusting for confounding factors in predicting inflammation, obesity, and PA were entered into the model together. PA still showed statistical significance in predicting inflammation, but the association between obesity and inflammation was no longer significant (see model 5). The odds ratio of associating PA to the risk of inflammation changed from 0.18 in model 2 (*p* = 0.006) to 0.18 in model 5 (*p* = 0.008). However, obesity no longer reached statistical significance in predicting inflammation. The odds ratio of associating obesity to the risk of inflammation changed from 2.59 in model 2 (*p* = 0.045) to 2.52 in model 5 (*p* = 0.062). This means the effect of PA on inflammation may reduce that of obesity on inflammation.

When adjusting for confounding factors in predicting depression, we included obesity, PA, and inflammation in the regression model. Inflammation still showed statistical significance in predicting depression (OR = 4.18, *p* = 0.004); however, the association between PA and depression were no longer significant (OR = 0.44, *p* = 0.124). On the other hand, the association between obesity and depression was still significant (OR = 2.45, *p* = 0.038) (see model 6). PA still showed statistical significance in predicting inflammation when obesity and PA were entered into the model together (see model 5). Therefore, inflammation may significantly mediate the relationships between PA and depression. Additionally, the mediating effect of inflammation (OR = 0.09, calculated in logits units and transformed to OR) tested significantly on the Sobel test (*z* = −2.01, *p* = 0.045).

## 4. Discussion

This study was aimed to determine the degree of association between depression and obesity and PA in older diabetes patients, mediated by inflammation. This study showed that older diabetics with depression had a lower intake of energy and protein, and a lower prevalence of smoking and alcohol consumption. In addition, our data indicated that depression and high inflammation was associated with PA levels and obesity status. Using binary logistic regression analysis, our data showed: (1) inflammation may only partially mediate the effect of obesity on increasing risk of depression; (2) inflammation may completely mediate the effect of PA on reducing risk of depression; (3) the effect of PA on reducing inflammation may lower that of obesity on increasing inflammation, and (4) inflammation may mediate the relationships between PA and depression, but not the relationships between obesity and depression by multiplication of its two sub-paths and Sobel test.

### 4.1. Relationship between Diabetes, Obesity, and Depression

Several studies have shown that there is a high prevalence of obesity among patients with type 2 diabetes [32,33,34,35]. According to the report of Nutrition and Health Survey in Taiwan (NAHSIT), the prevalence of obesity among older adults was 22.8% in 2013–2016 [36]. People with diabetes have a higher prevalence of obesity. According to some studies in Taiwan, the prevalence of obesity was 39.3 and 41.7% in diabetic men and women, respectively [34]. A study indicated that prevalence of obesity was 42.3% in Taiwanese women with type 2 diabetes [35]. In the present study, the prevalence of obesity was about 28.9% among elderly diabetes patients (men: 25.6; women: 31.8%).

Additionally, most recent studies have shown a higher prevalence of obesity in people with depression [37,38,39]. In the present study, older diabetes patients with obesity were 49.1% more likely to have depression compared with those who were non-obese (20.47%). Compared with people with a normal BMI, people with obesity had a higher rate of depression [38]. Data from the National Health and Nutrition Examination Surveys in the United States from 2005–2010 showed that adults with depression had a higher prevalence of obesity than adults without depression (43.2 vs. 33.0%) [36]. A cross-sectional study found that BMI was higher among overweight/obese patients who had depression [39].

Our results and several previous studies show that individuals with diabetes and obesity have an increased risk of depression in particular elderly patients [40,41]. Significant levels of depression can impact the quality of life and longevity in elderly people with diabetes. Therefore, the recommendations of positive health behaviors such as PA to prevent obesity and decrease the risk of depression are essential for these patients.

### 4.2. Relationship between Obesity, Inflammation, and Depression

Growing evidence suggests that obesity may increase the risk of depression [14,42]. Elevated levels of CRP are associated with an increased risk of depression [12]. In addition, studies have revealed the presence of obesity as the most significant determinant of low-grade chronic inflammation for unfavorable health effects [43,44]. Obesity and cognitive performance had a weak positive association in older people, partially mediated by inflammation [45]. The present study also indicated that depression was associated with obesity and inflammation. Moreover, our data showed that inflammation was a significant partial mediator of the relationship between obesity and depression.

A possible explanation for this finding is that adiposity may cause inflammation, which contributes to the development of depression [5]. In obesity, accumulation of free fatty acids activates pro-inflammatory serine kinase cascades, which in turn promotes adipose tissue to release interleukin-6 that stimulates hepatocytes to synthesize and secrete CRP [13,46,47]. Higher levels of inflammatory mediators found in many depressive individuals seem to interact with many of the pathophysiological aspects of depression, including neuroendocrine function, neurotransmitter metabolism, and synaptic plasticity [48]. This indicates that obesity could lead to an increase in inflammatory markers, which could increase the risk of depression [49]

The present study infers that obesity is associated with a higher risk of depression via an increase in inflammation. Older diabetes patients with obesity are recommended to lose weight through a healthy lifestyle that avoids triggering inflammation to reduce the risk of depression.

### 4.3. Relationship between PA, Inflammation, and Depression

Mounting evidence shows that inflammation may play an essential role in the development of depression [12,50]. Specific studies support that regular PA is associated with a decreased risk of depression and is used as a treatment for depression through the decrease in inflammation [51,52]. Similarly, the present study indicates that older diabetes patients with high PA levels have a lower rate of depression and high inflammation, whether older diabetes patients were obese or non-obese. Moreover, inflammation was a significant mediator of the relationships between PA and depression.

A possible explanation for this finding is that PA has a potent anti-inflammatory influence. There were significant effects of PA on inflammatory mediators produced within skeletal muscle, adipose tissue, and leukocytes, which promote an anti-inflammatory environment [52]. A prospective study revealed that reduced AGE-mediated inflammation may be related to PA-mediated increases in the circulating soluble receptor of advanced glycation end (AGE) products. This emphasis on the favorable health effects of PA on cardiovascular disease and other diseases is consistent with the decrease in inflammation [53].

Accordingly, this study infers that PA reduces risk of depression because of its capacity to reduce chronic low-grade inflammation. Regular PA is a critical lifestyle behavior that can prevent depression and chronic metabolic disease.

### 4.4. Relationship between Obesity, PA, and Inflammation

Several studies have suggested that PA can reduce inflammation, and obesity can trigger inflammation [44,52]. The present study indicates that obesity and PA are significantly associated with inflammation when obesity and PA were entered into a regression model, respectively. Specifically, obesity no longer had an effect on inflammation because the effect of PA on inflammation trumped that of obesity on inflammation when obesity and inflammation were entered into the model together. A possible explanation for this finding is that PA is a potent stimulus for reducing visceral adipose tissue, which may indirectly lower inflammation [52,54].

This study infers that the positive health effects of PA can reduce the inflammation produced by the adverse effects of obesity. Older patients with diabetes are advised to maintain an ideal weight with moderate PA and a healthy diet to reduce the risk of inflammation.

### 4.5. Relationship between Obesity, PA, Inflammation, and Depression

Although data from numerous studies suggest that obesity contributes to the inflammatory response, PA has anti-inflammatory effects that are associated with the risk of depression [12,14,48,51]. However, the strength of association between depression and obesity and PA in older diabetes patients, mediated by inflammation, is still unclear. The present study revealed that PA had no longer showed an effect on depression after controlling for the effect of inflammation. Thus, the unfavorable impact of inflammation on depression may be far greater than that of PA on depression when obesity, PA, and inflammation are considered in a regression model together. As PA still showed statistical significance in predicting inflammation, the association between obesity and inflammation was no longer significant when obesity and PA were entered into the model together. Additionally, the mediating effect of inflammation was significant according to the Sobel test.

Overall, this study speculates that the relationships between PA and depression among older diabetes patients are inflammation-mediated. Depression is highly prevalent among older patients with diabetes [55]. Moreover, older people with high inflammatory markers have an increased risk of depression and multiple chronic diseases [56]. Reducing the risk factors of developing inflammation is essential in preventing depression in older diabetes patients.

### 4.6. Achievements and Implications

Our results raise issues that may have policy implications for preventing and treating depression in older diabetes patients. It has been established that inflammation may play a critical role in developing depression [3]. Obesity could increase the risk of an inflammatory response, and the relationship between obesity and depression has also been documented [4]. PA was associated with a decreased risk of depression and as a treatment for depression because of its anti-inflammatory effects [51,52]. Nevertheless, very few studies explore how much of the association between depression, obesity, and PA in older diabetes patients is mediated through inflammation. The present study’s findings imply the beneficial effects of PA in reducing inflammation may reduce and ameliorate that of obesity and its effect of increasing inflammation. Inflammation may mediate the relationships between PA and depression. In future research, we should consider using PA to reduce the risk of developing inflammation to prevent and treat depression, especially for people with obesity.

### 4.7. Limitations and Prospective

Our study faced several limitations. First, this was a cross-sectional study. Though we found that the relation between the risk of depression to obesity, PA, and hsCRP showed different levels of mediation, we could not establish the exact causal relationship between predictors and depression. The relationship between predictors and depression should be validated by follow-up research. Further longitudinal studies are recommended. Secondly, dietary intake assessments and lifestyle behaviors relied heavily upon self-reported questionnaires. Patients may overestimate, underestimate, or forget their dietary intake and lifestyle behaviors. To overcome these limitations, the authors recommend that the assessment of dietary intake and lifestyle behaviors be done using more objective tools to reduce inaccuracy. Third, although this study has adjusted several of the confounding factors during data analytics projects, the model may have still omitted some potential vital information. Future research should consider other possible potential factors to reduce the uncertainty. Finally, the baseline of BMI is a simple measurement for adiposity. However, it cannot differentiate body fat or fat distribution. Future studies should consider waist circumference, hip circumference, and the waist-to-hip ratio.

## 5. Conclusions

This study infers that the positive health effects of PA can reduce the inflammation produced by the harmful effects of obesity. Overall, inflammation may be a mediator in the relationship between PA and depression in older diabetes patients. Older patients with diabetes should avoid becoming overweight or obese, maintain moderate and regular PA, adopt a healthy diet, and avoid smoking and excess alcohol consumption to reduce the risk of developing inflammation to prevent depression.

## Figures and Tables

**Table 1:** Characteristics of older adults with type two diabetes, according to depression and inflammation levels ^1^.

	Depression			Inflammation Levels			
Variables	With (*n* = 57)	Without (*n* = 140)	*p*	Low (*n* = 57)	Average (*n* = 105)	High (*n* = 35)	*p*
Age (years)	72.9 ± 6.2	71.8 ± 5.0	0.261	71 ± 4.3	72.4 ± 5.2	73.2 ± 6.8	0.124
Gender							
Men	13 (22.8)	77 (55.0)	<0.001	27 (47.4)	52 (49.5)	11 (31.4)	0.169
Women	44 (77.2)	63 (45.0)		30 (52.6)	53 (50.5)	24 (68.6)	
Education levels							
Primary school and below	51 (89.5)	124 (88.6)	0.712	49 (86.0)	95 (90.5)	31 (88.6)	0.396
Junior or senior high school	4 (7.0)	13 (9.3)		7 (12.3)	6 (5.7)	4 (11.4)	
University	2 (3.5)	3 (2.1)		1 (1.8)	4 (3.8)	-	
Duration of diabetes (years)	12.4 ± 7.9	10.5 ± 7.3	0.109	11.8 ± 7.8	10.8 ± 7.2	10.5 ± 8.1	0.618
Diabetes medication							
Oral hypoglycemic drug	36 (63.2)	102 (72.9)	0.178	43 (75.4)	73 (69.5)	22 (62.9)	0.435
Insulin and oral hypoglycemic drug	21 (36.8)	38 (27.1)		14 (24.6)	32 (30.5)	13 (37.1)	
Lipid-lowering medication	37 (64.9)	102 (72.9)	0.267	43 (75.4)	74 (70.5)	22 (62.9)	0.438
Hypertension medication	32 (56.1)	87 (62.1)	0.435	33 (57.9)	67 (63.8)	19 (54.3)	0.547
HbA1c	7.3 ± 1.2	7.3 ± 1.4	0.937	7.4 ± 1.3	7.2 ± 1.2	7.6 ± 1.5	0.169
eGFR	70.4 ± 18.5	71.5 ± 19.4	0.728	73.7 ± 20.5	72.6 ± 18.4	62.9 ± 17.0	0.017
Dietary intake							
Energy intake (Kcal/kg/day)	22.7 ± 5.9	27.0 ± 7.7	<0.001	26.3 ± 7.9	25.4 ± 6.5	25.9 ± 9.2	0.756
Protein intake (g/kg)	0.84 ± 0.30	0.71 ± 0.26	0.003	0.80 ± 0.28	0.80 ± 0.30	0.81 ± 0.33	0.999
Carbohydrate (% of energy)	61.0 ± 7.7	60.6 ± 8.9	0.748	61.6 ± 8.0	60.2 ± 9.1	60.6 ± 7.5	0.639
Fat (% of energy)	26.5 ± 6.1	26.9 ± 7.8	0.678	26.4 ± 6.8	27.0 ± 7.9	26.7 ± 6.3	0.897
Smoking							
Never smoked	50 (87.7)	93 (66.4)	0.006	41 (71.9)	75 (71.4)	27 (77.1)	0.031
Former smoked	6 (10.5)	29 (20.7)		3 (5.3)	16 (15.2)	-	
Currently smoking	1 (1.8)	18 (12.9)		13 (22.8)	14 (13.3)	8 (22.9)	
Alcohol consumption							
Never consumed	54 (94.7)	112 (80.0)	0.014	49 (86.0)	85 (81.0)	32 (91.4)	0.413
Formerly consumed	3 (5.3)	11 (7.9)		6 (10.5)	10 (9.5)	1 (2.9)	
Currently consuming	-	17 (12.1)		2 (3.5)	10 (9.5)	2 (5.7)	

^1^ The data that were presented in mean ± SD were analyzed by t-test. The data that were presented by number (*n*) and percent (%) were analyzed by chi-square tests. When cells had expected count less than five, data were analyzed by Fisher’s exact test. Statistically significant at *p* < 0.05.

**Table 2:** Prevalence of depression and high inflammation for combined PA levels with obesity status ^1,2^.

	PA Levels/Obesity Status						
Variables	A Group (Low/Obesity) (*n* = 19)	B Group (Moderate/Obesity) (*n* = 21)	C Group (High/Obesity) (*n* = 17)	D Group (Low/Non-Obesity) (*n* = 27)	E Group (Moderate/Non-Obesity) (*n* = 56)	F Group (High/Non-Obesity) (*n* = 57)	*p*
Depression							
With	12 (63.2)	11 (52.4)	5 (29.4)	11 (40.7)	11 (19.6)	7 (12.3)	<0.001
Without	7 (36.8)	10 (47.6)	12 (70.6)	16 (59.3)	45 (80.4)	50 (87.7)	
High inflammation							
With	9 (47.4)	6 (28.6)	-	5 (18.5)	10 (17.9)	5 (8.8)	0.001
Without	10 (52.6)	15 (71.4)	17 (100)	22 (81.5)	46 (82.1)	52 (91.2)	

^1^ The definition of obesity is as follows: BMI (kg/m^2^) ≥ 27. The definition of high inflammation is as follows: hsCRP (mg/L) of > 3. ^2^ Data were presented in number (*n*) and percent (%) and analyzed by chi-square tests. When cells had expected count less than five, data were analyzed by Fisher’s exact test. Statistically significant at *p* < 0.05.

**Table 3:** Relationships between physical activity, obesity, inflammation, and depression using binary logistic regression analysis.

Variable (*n*)	Model 1		Model 2		Model 3		Model 4		Model 5		Model 6	
	Depression ^1,2,3^		High Inflammation ^1,2,4^		Depression ^1,2,5^		Depression ^1,2,6^		High Inflammation ^1,2,7^		Depression ^1,2,8^	
	Odds Ratio (95% CI)	*p*	Odds Ratio (95% CI)	*p*	Odds Ratio (95% CI)	*p*	Odds Ratio (95% CI)	*p*	Odds Ratio (95% CI)	*p*	Odds Ratio (95% CI)	*p*
Obesity												
With (57)	2.91 (1.31–6.50)	0.009	2.59 (1.02–6.58)	0.045	2.54 (1.09, 5.78)	0.031			2.52 (0.96–6.61)	0.062	2.45 (1.05–5.70)	0.038
Without (140)	1.00		1.00		1.00				1.00		1.00	
Physical activity												
Low (46)	1.00		1.00				1.00		1.00		1.00	
Moderate (77)	0.49 (0.21–1.14)	0.098	0.61 (0.24–1.55)	0.302			0.52 (0.22–1.24)	0.140	0.66 (0.26–1.68)	0.379	0.53 (0.22–1.29)	0.161
High (74)	0.32 (0.12–0.86)	0.023	0.18 (0.05–0.61)	0.006			0.43 (0.16–1.20)	0.109	0.18 (0.05–0.64)	0.008	0.44 (0.16–1.25)	0.124
High inflammation												
With (35)	5.14 (2.01–13.13)				4.61 (1.77–12.00)		4.23 (1.37–13.08)				4.18 (1.56–11.20)	
Without (162)	1.00	0.001			1.00	0.002	1.00	0.012			1.00	0.004

^1^ For depression is a dependent variable, it was categorized as with/without depression to perform the binary logistic regression. When inflammation served as a dependent variable, it was categorized as high inflammation (greater than 3 mg/L of hsCRP) and non-high inflammation (the remainders) to perform the binary logistic regression. Statistically significant at *p* < 0.05. ^2^ Confounding factors were age, gender, eGFR, energy intake, protein intake, alcohol drinking, and smoking. ^3^ Obesity, physical activity levels and inflammation levels were entered into the model respectively. Confounding factors were adjusted. ^4^ Obesity and physical activity levels were entered into the model respectively and confounding factors were adjusted. ^5^ Obesity and inflammation levels entered model together and confounding factors were adjusted. ^6^ Physical activity and inflammation levels were entered into the model together and confounding factors were adjusted. ^7^ Obesity and physical activity levels were entered into the model together and confounding factors were adjusted. ^8^ Obesity, physical activity, and inflammation levels were entered into the model together and confounding factors were adjusted.

## Data Availability

The data are not publicly available due to privacy and ethical restrictions.

## References

[1] Wild S., Roglic G., Green A., Sicree R., King H. (2004). Global prevalence of diabetes: Estimates for the year 2000 and projections for 2030. Diabetes Care.

[2] Blay S.L., Fillenbaum G.G., Marinho V., Andreoli S.B., Gastal F.L. (2011). Increased health burden associated with comorbid depression in older Brazilians with diabetes. Affect Disord..

[3] Roy T., Lloyd C.E. (2012). Epidemiology of depression and diabetes: A systematic review. Affect Disord..

[4] Rustad J.K., Musselman D.L., Nemeroff C.B. (2011). The relationship of depression and diabetes: Pathophysiological and treatment implications. Psychoneuroendocrinology.

[5] Shelton R.C., Miller A.H. (2011). Inflammation in depression: Is adiposity a cause?. Dialogues Clin. Neurosci..

[6] Taylor L., Loerbroks A., Herr R.M., Lane R.D., Fischer J.E., Thayer J.F. (2011). Depression and smoking: Mediating role of vagal tone and inflammation. Ann. Behav. Med..

[7] Gea A., Beunza J.J., Estruch R., Sanchez-Villegas A., Salas-Salvado J., Buil-Cosiales P., Gomez-Gracia E., Covas M.I., Corella D., Fiol M. (2013). Alcohol intake, wine consumption and the development of depression: The PREDIMED study. BMC Med..

[8] Luciano M., Mottus R., Starr J.M., McNeill G., Jia X., Craig L.C., Deary I.J. (2012). Depressive symptoms and diet: Their effects on prospective inflammation levels in the elderly. Brain Behav. Immun..

[9] Song M.R., Lee Y.S., Baek J.D., Miller M. (2012). Physical activity status in adults with depression in the National Health and Nutrition Examination Survey, 2005–2006. Public Health Nurs..

[10] Howren M.B., Lamkin D.M., Suls J. (2009). Associations of depression with C-reactive protein, IL-1, and IL-6: A meta-analysis. Psychosom. Med..

[11] Patel A. (2013). Review: The role of inflammation in depression. Psychiatr. Danub..

[12] Hayashino Y., Mashitani T., Tsujii S., Ishii H. (2014). Elevated levels of hs-CRP are associated with high prevalence of depression in japanese patients with type 2 diabetes: The Diabetes Distress and Care Registry at Tenri (DDCRT 6). Diabetes Care.

[13] Rodriguez-Hernandez H., Simental-Mendia L.E., Rodriguez-Ramirez G., Reyes-Romero M.A. (2013). Obesity and inflammation: Epidemiology, risk factors, and markers of inflammation. Int. J. Endocrinol..

[14] Luppino F.S., de Wit L.M., Bouvy P.F., Stijnen T., Cuijpers P., Penninx B.W., Zitman F.G. (2010). Overweight, obesity, and depression: A systematic review and meta-analysis of longitudinal studies. Arch. Gen. Psychiatry.

[15] Hamer M., Hackett R.A., Bostock S., Lazzarino A.I., Carvalho L.A., Steptoe A. (2014). Objectively assessed physical activity, adiposity, and inflammatory markers in people with type 2 diabetes. BMJ Open Diabetes Res. Care.

[16] Rana J.S., Arsenault B.J., Despres J.P., Cote M., Talmud P.J., Ninio E., Wouter Jukema J., Wareham N.J., Kastelein J.J., Khaw K.T. (2011). Inflammatory biomarkers, physical activity, waist circumference, and risk of future coronary heart disease in healthy men and women. Eur. Heart J..

[17] Vallance J.K., Winkler E.A., Gardiner P.A., Healy G.N., Lynch B.M., Owen N. (2011). Associations of objectively-assessed physical activity and sedentary time with depression: NHANES (2005–2006). Prev. Med..

[18] Baron R.M., Kenny D.A. (1986). The moderator-mediator variable distinction in social psychological research: Conceptual, strategic, and statistical considerations. J. Personal. Soc. Psychol..

[19] Hayes A.F. (2022). Introduction to Mediation, Moderation, and Conditional Process Analysis: A Regression-Based Approach.

[20] Abu-Bader S., Jones T.V. (2021). Statistical Mediation analysis using the Sobel Test and Hayes Process Macro. Int. J. Quant. Qual. Res. Methods.

[21] Charan J., Biswas T. (2013). How to calculate sample size for different study designs in medical research?. Indian J. Psychol. Med..

[22] Wu C.S., Hsu L.Y., Wang S.H. (2020). Association of depression and diabetes complications and mortality: A population-based cohort study. Epidemiol. Psychiatr. Sci..

[23] Lee C.M., Chang C.F., Pan M.Y., Hsu T.H., Chen M.Y. (2017). Depression and Its Associated Factors among Rural Diabetic Residents. J. Nurs. Res..

[24] Tai S.Y., Ma T.C., Wang L.C., Yang Y.H. (2014). A community-based walk-in screening of depression in Taiwan. Sci. World J..

[25] Thompson F.E., Byers T. (1994). Dietary assessment resource manual. J. Nutr..

[26] American Psychiatric Association (1994). Diagnostic and Statistical Manual of Mental Disorders.

[27] Taiwan Health Promotion Administration, Ministry of Health and Welfare BMI Measurements. https://health99.hpa.gov.tw/onlineQuiz/bmi.

[28] Hu G., Qiao Q., Silventoinen K., Eriksson J.G., Jousilahti P., Lindstrom J., Valle T.T., Nissinen A., Tuomilehto J. (2003). Occupational, commuting, and leisure-time physical activity in relation to risk for Type 2 diabetes in middle-aged Finnish men and women. Diabetologia.

[29] Chodzko-Zajko W.J., Proctor D.N., Fiatarone Singh M.A., Minson C.T., Nigg C.R., Salem G.J., Skinner J.S. (2009). American College of Sports Medicine position stand. Exercise and physical activity for older adults. Med. Sci. Sports Exerc..

[30] Pearson T.A., Mensah G.A., Alexander R.W., Anderson J.L., Cannon R.O., Criqui M., Fadl Y.Y., Fortmann S.P., Hong Y., Myers G.L. (2003). Markers of inflammation and cardiovascular disease: Application to clinical and public health practice: A statement for healthcare professionals from the Centers for Disease Control and Prevention and the American Heart Association. Circulation.

[31] He J., Le D.S., Xu X., Scalise M., Ferrante A.W., Krakoff J. (2010). Circulating white blood cell count and measures of adipose tissue inflammation predict higher 24-h energy expenditure. Eur. J. Endocrinol..

[32] Chen L.I., Kuo M.C., Hwang S.J., Tsai J.C., Chen H.C. (2012). The Advantages and Drawbacks of Methods for Assessing Kidney Function in Clinical Practice. J. Intern. Med. Taiwan.

[33] AlShahrani M.S. (2021). Prevalence of obesity and overweight among type 2 diabetic patients in Bisha, Saudi Arabia. J. Family Med. Prim. Care.

[34] Tseng C.H. (2007). Body mass index and blood pressure in adult type 2 diabetic patients in Taiwan. Circ. J..

[35] Nfor O.N., Wu M.F., Lee C.T., Wang L., Liu W.H., Tantoh D.M., Hsu S.Y., Lee K.J., Ho C.C., Debnath T. (2018). Body mass index modulates the association between CDKAL1 rs10946398 variant and type 2 diabetes among Taiwanese women. Sci. Rep..

[36] Website of the Health Promotion Administration, Ministry of Health and Welfare Nutrition and Health Survey in Taiwan (NAHSIT) Report 2013–2016. https://www.hpa.gov.tw/EngPages/Detail.aspx?nodeid=1077&pid=6201.

[37] Pratt L.A., Brody D.J. (2014). Depression and obesity in the U.S. adult household population, 2005–2010. NCHS Data Brief.

[38] Pereira-Miranda E., Costa P.R.F., Queiroz V.A.O., Pereira-Santos M., Santana M.L.P. (2017). Overweight and Obesity Associated with Higher Depression Prevalence in Adults: A Systematic Review and Meta-Analysis. J. Am. Coll. Nutr..

[39] Sharafi S.E., Garmaroud G., Ghafouri M., Bafghi S.A., Ghafouri M., Tabesh M.R., Alizadehf Z. (2020). Prevalence of anxiety and depression in patients with overweight and obesity. Obes. Med..

[40] Gonzalez-Castro T.B., Escobar-Chan Y.M., Fresan A., Lopez-Narvaez M.L., Tovilla-Zarate C.A., Juarez-Rojop I.E., Ble-Castillo J.L., Genis-Mendoza A.D., Arias-Vazquez P.I. (2021). Higher risk of depression in individuals with type 2 diabetes and obesity: Results of a meta-analysis. J. Health Psychol..

[41] Chen F., Wei G., Wang Y., Liu T., Huang T., Wei Q., Ma G., Wang D. (2019). Risk factors for depression in elderly diabetic patients and the effect of metformin on the condition. BMC Public Health.

[42] Fulton S., Decarie-Spain L., Fioramonti X., Guiard B., Nakajima S. (2022). The menace of obesity to depression and anxiety prevalence. Trends Endocrinol. Metab..

[43] Monteiro R., Azevedo I. (2010). Chronic inflammation in obesity and the metabolic syndrome. Mediat. Inflamm..

[44] de Heredia F.P., Gomez-Martinez S., Marcos A. (2012). Obesity, inflammation and the immune system. Proc. Nutr. Soc..

[45] Gunathilake R., Oldmeadow C., McEvoy M., Inder K.J., Schofield P.W., Nair B.R., Attia J. (2016). The Association Between Obesity and Cognitive Function in Older Persons: How Much Is Mediated by Inflammation, Fasting Plasma Glucose, and Hypertriglyceridemia?. J. Gerontol. A Biol. Sci. Med. Sci..

[46] Rocha V.Z., Libby P. (2009). Obesity, inflammation, and atherosclerosis. Nat. Rev. Cardiol..

[47] Ellulu M.S., Patimah I., Khaza’ai H., Rahmat A., Abed Y. (2017). Obesity and inflammation: The linking mechanism and the complications. Arch. Med. Sci..

[48] Lee C.H., Giuliani F. (2019). The Role of Inflammation in Depression and Fatigue. Front. Immunol..

[49] Lotrich F.E., El-Gabalawy H., Guenther L.C., Ware C.F. (2011). The role of inflammation in the pathophysiology of depression: Different treatments and their effects. J. Rheumatol. Suppl..

[50] Raison C.L., Miller A.H. (2011). Is depression an inflammatory disorder?. Curr. Psychiatry Rep..

[51] Carek P.J., Laibstain S.E., Carek S.M. (2011). Exercise for the treatment of depression and anxiety. Int. J. Psychiatry Med..

[52] Nimmo M.A., Leggate M., Viana J.L., King J.A. (2013). The effect of physical activity on mediators of inflammation. Diabetes Obes. Metab..

[53] Sponder M., Campean I.A., Emich M., Fritzer-Szekeres M., Litschauer B., Graf S., Dalos D., Strametz-Juranek J. (2018). Long-term physical activity leads to a significant increase in serum sRAGE levels: A sign of decreased AGE-mediated inflammation due to physical activity?. Heart Vessel..

[54] Lee S., Kuk J.L., Davidson L.E., Hudson R., Kilpatrick K., Graham T.E., Ross R. (2005). Exercise without weight loss is an effective strategy for obesity reduction in obese individuals with and without Type 2 diabetes. J. Appl. Physiol..

[55] Park M., Reynolds C.F. (2015). Depression among older adults with diabetes mellitus. Clin. Geriatr. Med..

[56] Bektas A., Schurman S.H., Sen R., Ferrucci L. (2018). Aging, inflammation and the environment. Exp. Gerontol..

